# Supplementary figures and images for: Time-resolved dynamic computational modeling of human EEG recordings reveals gradients of generative mechanisms for the MMN response
Source: PLoS Comput Biol. 2023 Dec 13;19(12):e1010557. doi: 10.1371/journal.pcbi.1010557 (PMC10752554; doi:10.1371/journal.pcbi.1010557)

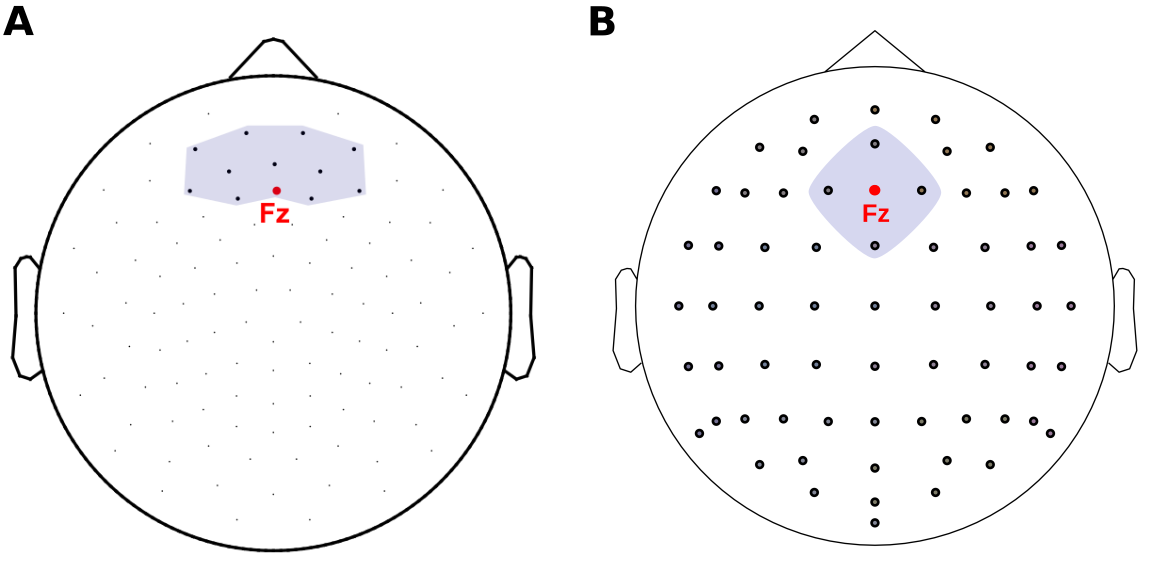

Supplement: S1 Fig — A: Study 1 EGI GSN200 system 128 electrodes layout, plotted on a head template. The frontal ROI is highlighted, comprising twelve frontal electrodes, including Fz. B: Study 2 Biosemi Active Two system 64 electrodes layout. For this study the selected ROI comprised only five frontal electrodes, also including Fz. (TIF) [file pcbi.1010557.s001.tif]

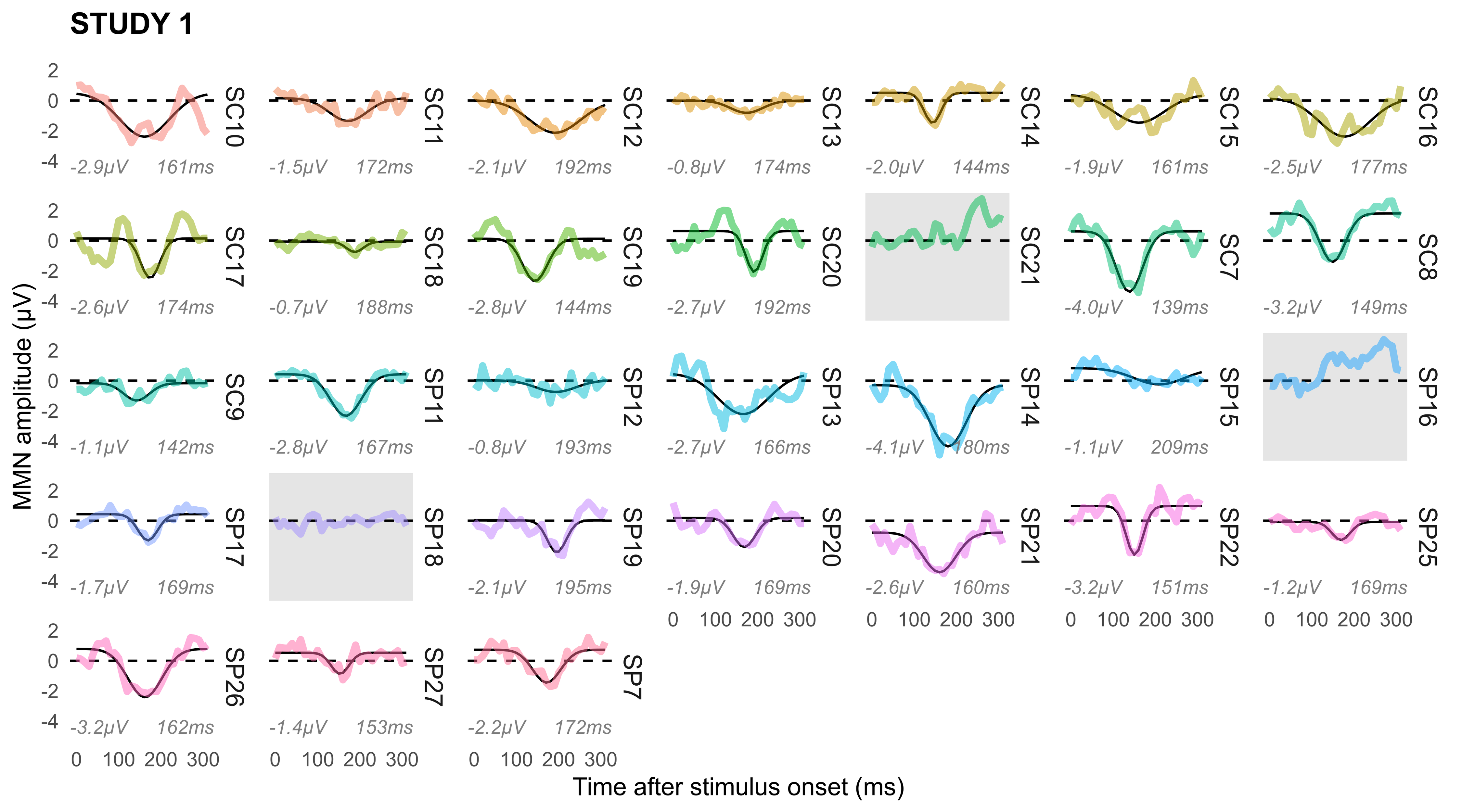

Supplement: S2 Fig — A Gaussian function has been fitted on each participant’s average ERP at the selected ROIs. Participants for whom the gaussian fit has failed to result in a negative peak (due to an absence of detectable MMN, or presence of a positive deflection instead of the expected negativity) have been excluded from subsequent analyses. (TIF) [file pcbi.1010557.s002.tif]

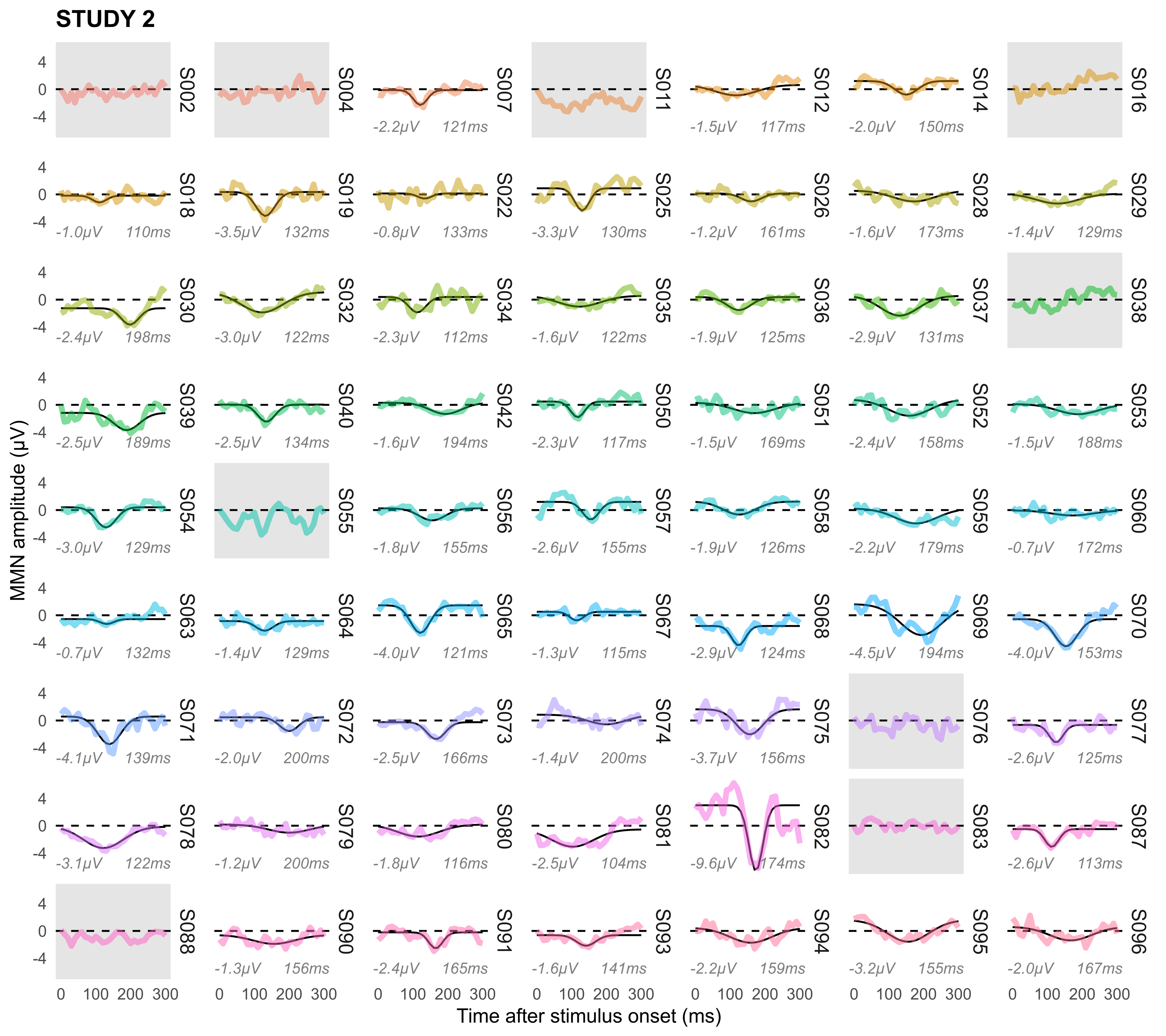

Supplement: S3 Fig — Same method as for Study 1. (TIF) [file pcbi.1010557.s003.tif]

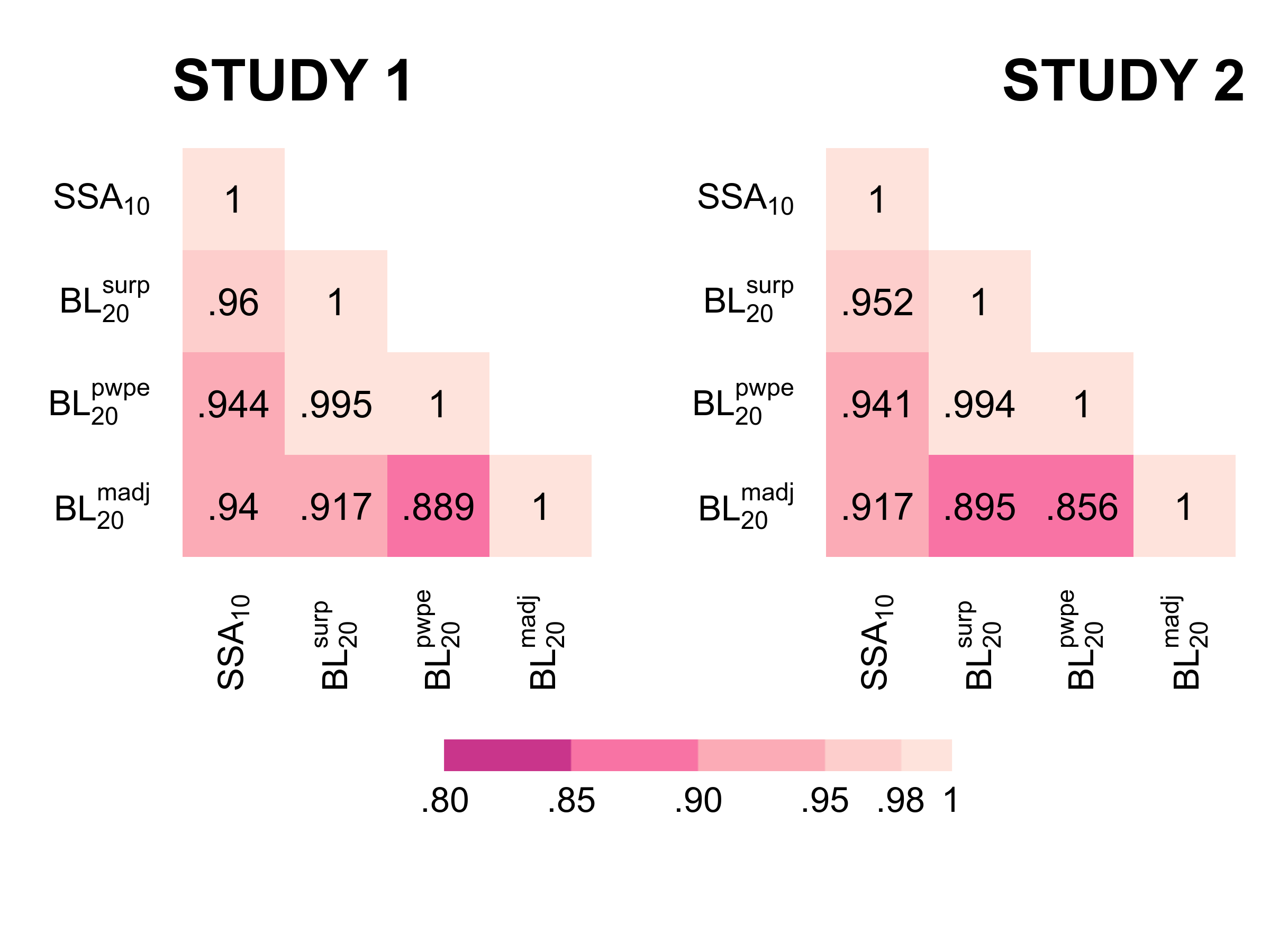

Supplement: S4 Fig — SSA: stimulus-specific adaptation, BL: Bayesian learning, surp: Shannon’s surprise, pwpe: precision-weighted prediction error, madj: model adjustment. (TIF) [file pcbi.1010557.s004.tif]

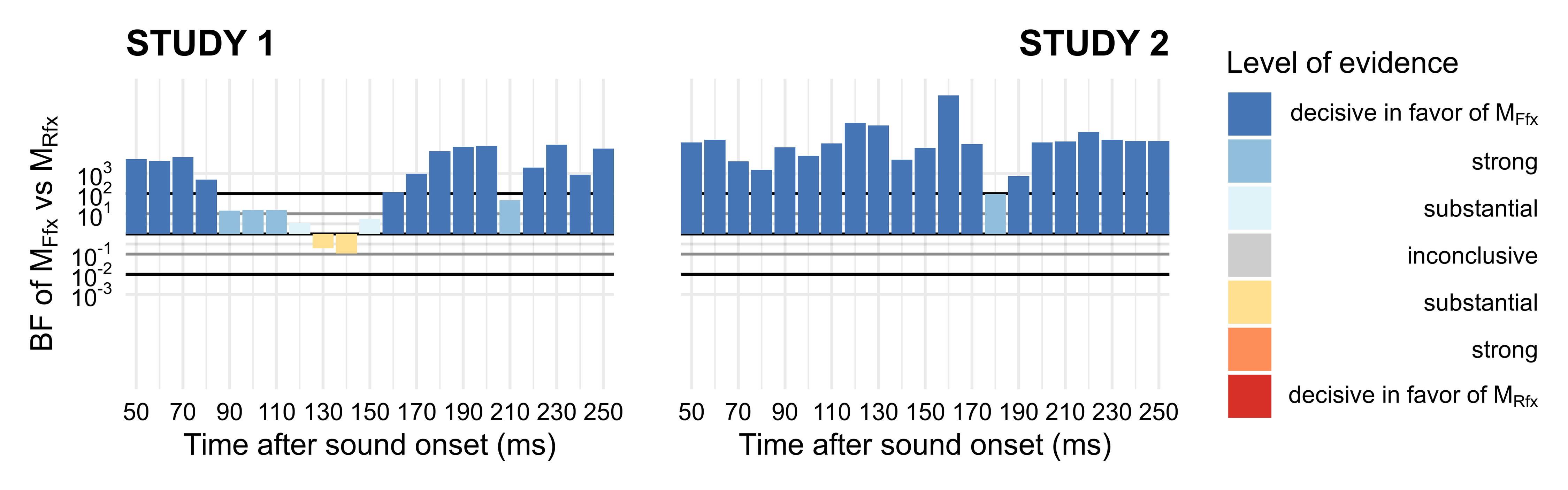

Supplement: S5 Fig — Model evidence for the two population structures underlying the ffx-BMC (homogeneous population, MFFX) and the rfx-BMC (heterogeneous population, MRFX) have been calculated and contrasted latency-wise at the level of model families. Only participants who displayed a negative MMN at the target latency were included. The homogeneous population distribution (MFFX) dominated over the entire MMN time-window in Study 2, and at most latencies in Study 1—except between 130 and 140ms. A closer inspection of models’ estimated frequencies at t = 130ms and 140ms in Study 1 indicated that the slight evidence in favor of population heterogeneity at these latencies is driven by BLmadj models competing with SSA models. Given that BLmadj models become dominant in the whole sample at 160ms, the apparent heterogeneity 20ms earlier might be attributed to inter-individual variation in the time at which the dominant generative mechanism of the MMN switches between adaptation and Bayesian-like processes. (TIF) [file pcbi.1010557.s005.tif]
