# Supplementary material for: Time-resolved dynamic computational modeling of human EEG recordings reveals gradients of generative mechanisms for the MMN response
Source: PLoS Comput Biol. 2023 Dec 13;19(12):e1010557. doi: 10.1371/journal.pcbi.1010557 (PMC10752554; doi:10.1371/journal.pcbi.1010557)
Supplement: S1 Text — (PDF) [file pcbi.1010557.s006.pdf]

Bayesian modeling of the population structure

The choice between a fixed-effect and a random-effect BMC may be arbitrated from data using Bayesian inference. Each of these approaches correspond to a specific *population model*: a Dirac and a multinomial distribution, respectively, which we will refer to M_{ffx} and M_{rfx}. These population models can be formalized in a Bayesian framework and their likelihood estimated and compared so as to infer the best population model given the observed subject-level models' evidence, the variational free energy F.
The assumption about the population structure can be formalized as a prior probability on the distribution of models frequencies in the population. Let us consider a set of K models m_k, each present in the population with a probability (or *frequency*) r_k, with $0 \leq r_k \leq 1$ and $\sum_{k=1}^{K} r_k = 1$. The vector $\mathbf{r}$ represents the distribution of models in the population and is unknown *a priori* but may can be adequately described using a multinomial, Dirichlet distribution. If, moreover, we have no prior assumption or information about models frequencies, we might reasonably assume them to be equally probable, which translates as a symmetric Dirichlet probability density function for the prior on $\mathbf{r}$:

$$p(\mathbf{r}) = Dir(\alpha) = \frac{\Gamma(K\alpha)}{\Gamma(\alpha)^K} \prod_{k=1}^{K} r_k^{\alpha-1} \qquad (1)$$

where Γ is the gamma function and α a concentration parameter that controls the degree of heterogeneity of the population.
When $\alpha \gg 1$ the prior strongly favors equiprobable distributions of models in the population, thus dismissing poorly heterogeneous population hypothesis. However, the heterogeneous population assumption is more appropriately formalized as an uninformative (flat) prior that is uniform over the space of possible distributions of models frequencies, which can be obtained with $\alpha = 1$. On the other hand, when α tends towards 0 the prior on $\mathbf{r}$ favors population distributions that are concentrated around a unique model, i.e. a homogeneous population. Rigoux et al. (2014) derived approximations of population model evidence; we refer the readers to their paper for mathematical details [1]. These measures have been implemented in the VBA toolbox and are provided by the "VBA_groupBMC" function.

References

[1] Rigoux L, Stephan KE, Friston KJ, Daunizeau J. Bayesian Model Selection for Group Studies - Revisited. Neuroimage. 2014;84:971–985. doi:10.1016/j.neuroimage.2013.08.065.
