# Supplementary material for: Time-resolved dynamic computational modeling of human EEG recordings reveals gradients of generative mechanisms for the MMN response
Source: PLoS Comput Biol. 2023 Dec 13;19(12):e1010557. doi: 10.1371/journal.pcbi.1010557 (PMC10752554; doi:10.1371/journal.pcbi.1010557)
Supplement: Attachment — Submitted filename: MMN_modeling_review_response.pdf [file pcbi.1010557.s007.pdf]

MMN modeling - PLOS Comput Biol Review response 07/11/23

We thank the reviewers for their time reviewing our manuscript and for their positive overall reply. Please find below our detailed point-by-point reply. The most important changes we made to the manuscript are quoted here and are also highlighted in red in the revised manuscript. Other minor additions independent of the reviewer's comments can also be seen in red.

Reviewer #1

This is an excellently written and informative paper on the putative computational mechanisms of mismatch negativity, a classical neural response which has been linked to adaptation, deviance detection, and Bayesian surprise/learning. The authors disambiguate between these different interpretations in a model-based time-resolved analysis of two large EEG datasets. The manuscript will be of interest for a large audience of cognitive, computational, and systems neuroscientists.

1. The introduction is very well written - however, I would encourage authors to also discuss more recent computational work on somatosensory and multimodal mismatch responses, which does differentiate between early and late electrophysiological signatures of surprise (dois: 10.1371/journal.pcbi.1008068 and 10.1101/2022.10.27.514010)

>> We would like to express our gratitude to the reviewer for suggesting these very interesting articles that we were surprisingly not aware of. We are glad to see that independently using similar models and analysis methods as them, but within a different sensory modality, we reach somehow converging conclusions about the temporality of generative mechanisms of the MMN. We added the references in the section of the introduction listing the computational works on mismatch responses (section *Insights from computational modeling*) which now reads like this:

“Recent work extended the [Bayesian learning] model to include different neural surprise signatures and could show interesting spatial and cortical dynamics of the somatosensory mismatch response [Gijsen et al,. 2021]. Similar computational models were also applied to the auditory MMN in fused EEG-MEG [Lecaignard et al., 2021] and ECoG [Lecaignard et al., 2022] measurements, and to the visual MMN in EEG [Stefanics et al., 2018], confirming the previous results and designing new Bayesian learning models of deviance detection. Finally, Grundei et al. very recently showed evidence for Bayesian learning against classical change detection to explain cross-modal EEG mismatch responses (auditory, somatosensory and visual) in humans [Grundei et al., 2023].”

And also used their results to enrich our discussion (section *Multiple mechanisms may underlie the MMN response at different latencies*):

“Differently, a recent computational modeling study of the somatosensory mismatch revealed partial evidence for a dominance of model adjustment BL process (there called "Bayesian surprise") from 150 to 200 ms both at scalp and source levels [Gijsen et al., 2021], reminiscent of our own results, although they did not test any adaptation-like models. Instead, at earlier latencies their data were better explained by a "confidence corrected surprise" BL model that we did not include in our analysis. We leave it for future studies to disambiguate between adaptation and "confidence corrected surprise" BL mechanisms during the early window of the MMN. It is worthwhile to note that the same authors further showed the involvement of Bayesian learning mechanisms in the generation of the MMN for three different sensory modalities (auditory, somatosensory and visual), as well as a signature of cross-modal processing in a later mismatch component (P3a, 300-350 ms) [Grundei et al,. 2023].”

2. I think the use of the word “declined” in the sentence in line 104 is confusing; perhaps a better alternative would be “included”, “nuanced”, “disambiguated” etc.

>> Thank you for pointing this out, we followed your suggestion and changed it to “nuanced” in the revised manuscript.

3. Methods: The filtering settings (double high-pass filter, first at 0.5Hz, then at 1Hz) need to be revised and/or justified in light of previous analyses showing that such high cut-off frequencies introduce strong distortions in ERP amplitudes and latencies (e.g., doi: 10.3389/fpsyg.2012.00131) . This is even more problematic for study 2, where a 2-Hz high-pass filter was used.

>> This is an important point that is indeed underestimated in EEG studies. The filtering settings implemented in the present study are those of the initial publications. For the second study, the use of a 2-Hz high-pass filter was required by the presence of low frequency artifacts presumably caused by abundant sweating due to the threat-induction procedure of the paradigm, as described in the manuscript and in the original article presenting these data [1]. The use of rather strong high-pass filtering was further justified in the context of modeling trial-to-trial variations by the need to remove signal drifts in the spectral range corresponding to interstimulus interval and below (0.5-1 Hz). On the other hand, we are not comparing experimental conditions where spurious effects may be produced by filters on differences of peak amplitudes or latencies. More importantly, the absence of a P3 component in our passive oddball paradigms reduces considerably the risk of strong distortions of earlier components by zero-phase (non-causal) filters, even at cut-off frequencies as high as 1.5Hz, as can be seen in figure 7A of Widmann et al. 2015 [3].

We added a caution statement in the *Perspectives* section:

“We would also like to acknowledge some limitations of the current study. First, the EEG datasets we used from previous publications were submitted to rather aggressive high--pass filtering settings. As explained in the Methods section, the use of a 2-Hz high-pass filter in the second study was necessary to remove low frequency artifacts caused by

threat-induction. For both studies however, in the context of trial-wise modeling, strong high-pass filtering also permitted to remove signal drifts in the spectral range of interstimulus interval and below (0.5-1 Hz). Finally our oddball paradigm being passive, no P3 component was elicited, which considerably reduces the risk of distortions of earlier components by zero-phase (non-causal) filters as previously shown with high-pass at 1.5-Hz and above [Widmann et al., 2015]. Nevertheless, we would advise future replications to use lower high-pass filtering thresholds, when possible, as recommended by recent guidelines on the topic [Widmann et al., 2015; Decheveigne & Nelken 2019]"

4. The choice to only model ERPs in sensor-space is a limitation of the study. The study would benefit from a more direct analysis of separation between hierarchical levels and/or time scales of adaptation based on source reconstruction of ERPs. This is to some extent addressed in the discussion, but the datasets presented here yield a high potential to clarify some of the controversies regarding the source-level implementation of MMN present in the literature. I would encourage the authors to either include such an analysis in the paper, or discuss the reasons for not including it.

>> We agree that our results naturally call for an extension in the spatial domain in order to complete the description of the neurocomputational architecture underlying the generation of the MMN. In the "Perspectives" section we suggested that future replication studies should include a spatial dimension. In particular, they should be optimally designed to maximize the ability to localize and dissociate spatial sources of neuroelectric signals. The need for an optimized methodology is exacerbated in the context of the auditory MMN, which has been shown to be generated by multiple, anatomically close cortical structures [2–4]. Unfortunately, the EEG datasets used in the current study were not acquired with source reconstruction analyses in mind and therefore individual electrode positions were not recorded. While it is technically possible to perform source reconstruction without this information using generic templates, the result would likely be too coarse to disentangle two neighboring sources (~10mm in the context of auditory MMN), yielding a high risk for false negative results.

We acknowledged this in the last paragraph of the *Perspectives* section which now reads:

"Additionally, we could not implement cortically resolved modeling in this study given the EEG datasets we used were not optimized for source reconstruction analyses. We encourage future work to refine the mechanistic accounts of the MMN by fitting time-resolved models on data with high spatial resolution such as MEG- or fused EEG/MEG-derived cortical sources [Lecaignard et al,. 2021], as well as by testing models that are more biophysically plausible [...]."

5. What was the reason for constructing several SSA models with fixed adaptation parameters rather than fitting these adaptation parameters to the data? Similarly, could forgetting constants be fitted to the data in case of BL models?

>> Indeed past modeling studies have adopted two distinct approaches to define the model set: a first one we could call "structural" where individual models are differentiated by their structure, with model-specific parameters being estimated in the model fitting, and what we

could call a "parametric approach" where each distinct model structure is instantiated in multiple versions differing only in their values of the model parameters (the approach we chose for our study). When exploring the range of parameter values with individual models is intractable—typically when models have more than one parameter, as in DCM studies—the structural approach is well suited to describe the model space. However, the structural approach implicitly assumes that the generative mechanism has a unique set of parameter values (which may be individual-specific). Here, in the context of the MMN, we could not exclude the possibility that multiple models, with a similar computational architecture but differing in parameter values, generated the data in parallel i.e. at the same latency. Animal studies, for example, have shown that neural adaptation happens simultaneously at distinct cortical areas but at different adaptation rates [5]. Therefore, we reasoned that a parametric approach, where each putative mechanism is implemented in a model duplicated in multiple versions with distinct parameter values, was more appropriate for the problem we tackled.

6. The text in line 275 mentions six SSA models but there are seven taus in line 276. Could the authors clarify this discrepancy?

>> This is a typo, it should read "seven", thank you for pointing this out.

7. Line 280, typo: "We inspired" - should be "We were inspired"?
8. Between lines 289-290, typo: "Ajustment" - should be "Adjustment"

>> Thank you for pointing out these typos.

9. While the model recovery analysis is a valuable part of the manuscript, I wasn't convinced by limiting it to one set of taus, especially given the fact that the authors explicitly consider a wide range of fixed taus when fitting their models (see point 5). It would be good to see a more complete recovery analysis (with a range of taus), and/or a specific recovery analysis focusing on recovering parameters (taus) rather than models.

>> As far as we understand it, this comment offers two distinct suggestions: a) extending the model confusion analysis to a wider range of taus, and b) complementing it with a parameter recovery analysis.
In our opinion, these two analyses address fairly different questions. A confusion analysis estimates the ability of the experimental design and data to differentiate between structurally distinct generative models. This analysis requires fixed parameter values for each model. Exploring a wide range of parameter values in such an analysis mixes the structural and parametric levels, complicating its interpretation. In addition, simulation studies are computationally very demanding as they require a large number of repetitions (in the order of 100-1000) to obtain reliable estimates, an issue that is exacerbated in confusion analyses by a quadratic dependency of computational cost on the size of the model space. Our strategy was to run simulations with one parameter value only per generative model, chosen carefully to 1) be representative of what was actually observed in the model fits on real data and 2) be conservative in the sense of having the highest risk for model confusion. The actual choice was motivated in the first paragraph of section *Model recovery* in the *Methods*.

On the other hand, a parameter recovery analysis characterizes the ability of the experimental design and measures to accurately identify the true data-generating parameter

values. Issues of parameter identifiability arise in multi-parameter models where distinct combinations of parameters' values may generate the exact same observed outcome. A well-known example is provided by the drift-diffusion model which, in its complete form, is invariant to parameter scaling and therefore requires further simplifying assumptions to be properly estimated [6]. Our study tests single-parameter models only, which were designed to generate the phenomenon of interest, and are therefore necessarily identifiable.

10. In discussing their absolute goodness of fit, the authors may want to refer to a paper (albeit not based on MMN) where similar R2 values have been found (doi: 10.7554/eLife.11476.001)

>> Thank you for this suggestion, it is quite interesting that we obtain similar R2 values while this study used LFP data. We added it while presenting our goodness of fit results:

"While these figures may seem low at first glance they are consistent with previous results (1% of LFP data variance explained in [Sedley et al,. 2016])"

11. In discussing the possibility that different MMN latencies reflect different mechanisms, the authors may want to refer to a paper which showed that early latencies reflect deviance processing itself while late latencies reflect deviance feature processing (doi: 10.3389/fnhum.2021.613903), suggesting a difference in the levels of abstraction between early and late responses.

>> The reviewer points towards an interesting study which attempted to compare the MMN responses to different acoustic features [7]. While we agree with the validity of their hypothesis within the predictive coding framework, we also think that their results show only partial evidence for a temporal dissociation between deviance processing per se and deviance feature processing. Indeed their univariate ERP analysis revealed no difference in the MMN response to various acoustic features, consistently with previous results [3], and only a secondary multivariate decoding analysis showed a specificity of deviance features but at unusually late latencies (> 250ms) as they acknowledge themselves. We would therefore prefer to remain careful with quoting these somewhat inconsistent results and will prefer to wait for their further replication. Trial-wise modeling of neural data from multi-features paradigms would be a particularly interesting example to address this question.

12. In discussing the work of Garrido in the context of backward connections, another piece of evidence comes from their 2009 paper (doi: 10.1016/j.neuroimage.2009.06.034), where adaptation has been linked to modulation of intrinsic connectivity (gain parameters) while model learning has been linked to modulation of extrinsic (e.g., backward) connectivity.

>> We did not discuss this particular paper because the model used was less sophisticated than other efforts from the same team (as ascertained by the authors themselves); in particular they modeled dynamics in *forward* extrinsic connections only, not backward connections. Moreover, the link between adaptation and intrinsic connectivity on one hand, and between model learning and changes in extrinsic connectivity on the other hand, is a framing of their results by the authors rather than a result *per se* because adaptation and model learning are not modeled computationally as in our study and others.

Reviewer #2

Poublan-Couzardot and co-authors tested in their manuscript entitled 'Time-resolved dynamic computational modeling of human EEG recordings reveals gradients of generative mechanisms for the MMN response' different computational models and fitted these to EEG-Date to reveal the underlaying mechanism of the MMN. This is another important step in the attempt in recent years through model-based analysis techniques to better describe the dynamics of brain signals for mismatch responses. The manuscript is clearly written and the analyses and results are quite easy to follow. However, to enable other scientists to perform the analyses on their data as well, it would be nice if the authors described the individual steps with the VBA toolbox in more detail.

>> We tried to strike a balance between describing the modeling framework and the parameters critical for replication (e.g. priors) without diving too much into the details of the VBA toolbox so as not to overload the Methods section which is already lengthy. The Matlab code used for the modeling will been made openly accessible and linked to in the Data Availability statement of the publication so that other scientists can reuse it for their analyses, here is the repository:
https://osf.io/dxj5p/?view_only=c6ecc9a450184d31bcb80268ab029be4

The other point that is important to me is that the analysis should not only be performed on pre-selected electrodes. I think it is important to show not only the temporal effects but also the spatial specification of the results since it is conceivable that different models are dominating over different brain regions. I know that this is computationally expensive and that the results are not easy to present, but it is possible and would add significant scientific value to the results from my point of view (please see for a similar approach Gijsen et al., PloS Comput Biol. 2021). If the manuscript becomes too long due to the additional analysis of the whole electrode space, from my point of view the simulations could be taken into the supplement.

>> While we completely agree that different generative models are likely to be not only temporally, but also spatially segregated, it seems to us that the auditory MMN is not well suited for this endeavor as the topography of the MMN is very stable across the entire time-window. This is in contrast with what has been reported in the somatosensory domain, e.g. by Gijsen and colleagues, and is further exemplified in a more recent article from the same research team studying simultaneously auditory, somatosensory and visual mismatch responses [8]. It can be seen in Figure 3 that the auditory MMN fronto-central cluster is quite stable from 80 to 200 ms, while the somatosensory MMN starts at fronto-central electrodes around 50 ms and then shifts to a bilateral temporal cluster between 90 and 190 ms. Yet in all modalities, during the time of the MMN, their modeling results suggest a homogeneity of surprise read-outs across the whole electrode space (Figure 6b). An even more interesting analysis would have been to apply our modeling framework to cortical sources space, however, as we explained in a response to reviewer 1 above, the EEG datasets we used were not optimized for source reconstruction and we decided to not implement it.

Minor:
The title should better reflect the results of the study.

>> In our opinion, the two important results of the study are 1) the temporal dissociation between SSA and BL models, and 2) the gradient in adaptation rate for SSA. We could not find a satisfying title that would reflect both results parsimoniously, without sacrificing important information pertaining to the methodological approach. Trying to tick all boxes results in a lengthy title such as: *Time-resolved dynamic computational modeling of human EEG recordings reveals a temporal dissociation between generative mechanisms for the MMN response and a gradient of stimulus-specific adaptation rates*
We would be happy to have the opinion of the editor on this issue.

It was unclear to me why the model was not fitted until 45 ms. One would certainly not expect large effects in this time, but it would contribute to the completeness of the analysis.

>> We initially fitted the models from 0 to 300 ms but decided to reduce it to 50-250ms as indeed no significant trial-to-trial dynamics were found outside of this interval. On a more theoretical note, while the classical auditory ERP does include a positive deflection around 50ms, it is rather weak on EEG signals due to the depth of the neural sources, and more importantly it is not sensitive to stimulus deviance as indicated by the absence of significant signal in the ERP differences reported by MMN studies that we are aware of. As can be seen in our results the null model already outcompetes all others below 80ms and above 190 or 220ms (depending on the study, see Figure 7). Yet it is worth noting that this interval extends to both earlier and later latencies when the analysis is restricted to participants with very high signal-to-noise ratio (figure 8), but there were too few of such participants to reach conclusions at the earliest latencies (1 in study 1, 3 to 5 in study 2).

We added the explanatory note in the *Model Fitting* section of the Methods:

"We restricted the modeled interval to 50-250ms so as to alleviate the computational burden while covering the time window of statistical significance of the MMN usually found in the literature (100-200ms)."

The average over 10 ms also seems quite long to me. Despite the additional computing time, the authors should consider increasing the temporal resolution, e.g. 5 ms.

>> The choice of a 10ms step for the temporal resolution of the analysis was dictated by the 60Hz cutoff threshold of the low-pass filter applied. Such filtering is standard in EEG preprocessing and takes into account the fact that most signal power lies below 40Hz in ERP studies [9]. Therefore, a smaller time step for modeling is unlikely to yield finer temporal information.

Reviewer #3

This paper details a very thorough and yet accessible approach to assessing which of a series of models can best account for changed responsiveness to auditory stimuli in an oddball sequence. The results show quite convincingly that different processes are likely to be most influential at different time points within the auditory event-related potential.
The paper is exquisitely written. The material is complex for a reader from a non-computational background but the way it is presented, and the thoroughness of the

approach, makes this a very informative and convincing article. I have no major issues and only a few very minor comments that the authors might consider.

p20 line 302. Is the prior mean you refer to here implemented at an individual level?
>> Yes indeed. We have clarified this point in the revised manuscript.

Figure 2 - although in text you say there isn't much to glean from the BL measures it does seem that where there is a tendency to outperform others it seems always to be 20-50ms. Is this worth noting?
>> Commenting Figure 2, we have chosen to highlight the most significant influences of the tau parameter, which are found for BLmadj and SSA. While it is true that the evidence for BLpwpe depends slightly on tau, it is to a much lesser extent than other models and with some inconsistency between the two studies (e.g. BLpwpe10 has the highest peak of evidence in study 1 but is rather low in study 2).

I think the data displays you have chosen are quite spectacular in communicating the outcomes of the analyses. It was a pleasure to read this paper and I believe it will be an excellent contribution to the literature.
>> Thank you for this supportive comment, we have tried our best to communicate these complicated results.

References

1. Fucci E, Poublan-Couzardot A, Abdoun O, Lutz A. No effect of focused attention and open monitoring meditation on EEG auditory mismatch negativity in expert and novice practitioners. Int J Psychophysiol. 2022;176: 62–72. doi:10.1016/j.ijpsycho.2022.03.010
2. Lecaignard F, Bertrand O, Caclin A, Mattout J. Empirical Bayes evaluation of fused EEG-MEG source reconstruction: Application to auditory mismatch evoked responses. NeuroImage. 2021;226: 117468. doi:10.1016/j.neuroimage.2020.117468
3. Phillips HN, Blenkmann A, Hughes LE, Bekinschtein TA, Rowe JB. Hierarchical Organization of Frontotemporal Networks for the Prediction of Stimuli across Multiple Dimensions. J Neurosci. 2015;35: 9255–9264. doi:10.1523/JNEUROSCI.5095-14.2015
4. Phillips HN, Blenkmann A, Hughes LE, Kochen S, Bekinschtein TA, Cam-CAN, et al. Convergent evidence for hierarchical prediction networks from human electrocorticography and magnetoencephalography. Cortex. 2016;82: 192–205. doi:10.1016/j.cortex.2016.05.001
5. Nieto-Diego J, Malmierca MS. Topographic Distribution of Stimulus-Specific Adaptation across Auditory Cortical Fields in the Anesthetized Rat. Zatorre R, editor. PLOS Biol. 2016;14: e1002397. doi:10.1371/journal.pbio.1002397
6. Ratcliff R, Smith PL, Brown SD, McKoon G. Diffusion Decision Model: Current Issues and History. Trends Cogn Sci. 2016;20: 260–281. doi:10.1016/j.tics.2016.01.007
7. An H, Ho Kei S, Auksztulewicz R, Schnupp JWH. Do Auditory Mismatch Responses Differ Between Acoustic Features? Front Hum Neurosci. 2021;15. Available: https://www.frontiersin.org/articles/10.3389/fnhum.2021.613903
8. Grundei M, Schröder P, Gijsen S, Blankenburg F. EEG mismatch responses in a multimodal roving stimulus paradigm provide evidence for probabilistic inference across audition, somatosensation, and vision. Hum Brain Mapp. 2023;44: 3644–3668. doi:10.1002/hbm.26303
9. Widmann A, Schröger E, Maess B. Digital filter design for electrophysiological data - a practical approach. J Neurosci Methods. 2015;250: 34–46. doi:10.1016/j.jneumeth.2014.08.002
